# Supplementary material for: Reproducible analysis of disease space via principal components using the novel R package syndRomics
Source: eLife. 2021 Jan 14;10:e61812. doi: 10.7554/eLife.61812 (PMC7857733; doi:10.7554/eLife.61812)
Supplement: Source code 1. [file elife-61812-code1.zip › AnalysisscriptforsyndRomics.html]

Analysis script for: Reproducible analysis of disease space via principal components: a brief tutorial and R package (syndRomics)


# Analysis script for: Reproducible analysis of disease space via principal components: a brief tutorial and R package (syndRomics)

#### Torres-Espin A, Chou A, Huie JR, Kyritsis N, Upadhyayula PS, Ferguson AR

#### 15/12/2020

# Intro

This script contains the analysis of 2 case studies that demonstrate the use of syndromics analysis and the syndRomic package. Details of these can be seen in the paper:

```
Abel Torres-Espin, Austin Chou, J. Russell Huie, Nikos Kyritsis, Pavan Upadhyayula, and Adam R. Ferguson. Reproducible analysis of disease space via principal components: a brief tutorial and R package (syndRomics).
```

**Libraries**

Libraries used in this notebook:

```
library(syndRomics)
library(tidyverse)
library(mice)
library(MissMech)
library(naniar)
library(progress)
library(Gifi)
```

If these packages are not present, they can be installed with:

```
install.packages("packageName")
```

syndRomics package can be installed from GitHub using the following code:

```
install.packages('devtools')
devtools::install_github(repo = "ucsf-ferguson-lab/syndRomics@*release")
```

This will install the latest release. To install previous versions, you can use:

```
devtools::install_github(repo = "ucsf-ferguson-lab/syndRomics@versionNumber")
```

For example:

```
devtools::install_github(repo = "ucsf-ferguson-lab/syndRomics@0.0.1.9000")
```

# First case study

The data comes from Open Data Commons for Spinal Cord Injury (ODC-SCI) dataset 26. The same data have been used in the past by Adam Ferguson team to perform SCI syndromics analysis:

```
Ferguson AR, Irvine K-A, Gensel JC, Nielson JL, Lin A, Ly J, et al. Derivation of Multivariate Syndromic Outcome Metrics for Consistent Testing across Multiple Models of Cervical Spinal Cord Injury in Rats. PLOS ONE. 2013 Mar 27;8(3):e59712.
```

10.1371/journal.pone.0059712

For details on the analysis, please see the manuscript.

## Data pre-processing

Loading the data and extracting the variables at 6 weeks after injury. To re-run the script, you will need to download the data and change the path in the code.

```
# Specify the path and file name of the ODC-SCI:26 data from odc-sci.org
odc.df<-read.csv('data/odc-sci_26.csv', na.strings = '')
odc.df<-odc.df[-1,]

# select variables at week 6 and shorten the variable name
odc.df.filter<-odc.df[,str_detect(colnames(odc.df),
                                  "_wk6")]
colnames(odc.df.filter)<-str_remove(colnames(odc.df.filter), "_wk6")
colnames(odc.df.filter)<-str_remove(colnames(odc.df.filter), "AbsDev")
```

## Missing data analysis and imputation

We first visualize the missingess and the missing patterns.

```
set.seed(500)

#Shadow plot
vis_miss(as.data.frame(odc.df.filter))+
  theme(plot.margin = unit(c(3,5,3,3), 'mm'),
        legend.position = 'right')
```

```
ggsave('Case1 missing shadow.png', width = 5, height = 4)

#Upset plot
upset<-naniar::gg_miss_upset(as.data.frame(odc.df.filter),nsets=20, mb.ratio=c(0.4,0.6))

upset
```

```
png('Case1 missing upset.png', units = 'in', width = 3.5, height = 3.5, res = 300)
  upset
dev.off()
```

```
## png 
##   2
```

Now we test whether the data is missing complete at random (MCAR).

```
odc.df.filter<-as.matrix(apply(odc.df.filter, 2, as.numeric))
odc.df.filter%>%TestMCARNormality()
```

```
## Call:
## TestMCARNormality(data = .)
## 
## Number of Patterns:  3 
## 
## Total number of cases used in the analysis:  157 
## 
##  Pattern(s) used:
##           wtChng   RFSL   RFPA   StepDistRF   LFSL   LFPA   StepDistLF   RHSL
## group.1        1     NA     NA           NA     NA     NA           NA     NA
## group.2        1     NA     NA           NA     NA     NA           NA     NA
## group.3        1      1      1            1      1      1            1      1
##           RHPA   StepDistRH   LHSL   LHPA   StepDistLH   Groom   PawPL
## group.1     NA           NA     NA     NA           NA       1       1
## group.2     NA           NA     NA     NA           NA       1       1
## group.3      1            1      1      1            1       1       1
##           BBB_FergTrans   TotalSubscore   ForelimbOpenField   Number of cases
## group.1              NA               1                   1                 7
## group.2              NA              NA                  NA                26
## group.3               1               1                   1               124
## 
## 
##     Test of normality and Homoscedasticity:
##   -------------------------------------------
## 
## Hawkins Test:
## 
##     P-value for the Hawkins test of normality and homoscedasticity:  2.251257e-21 
## 
##     Either the test of multivariate normality or homoscedasticity (or both) is rejected.
##     Provided that normality can be assumed, the hypothesis of MCAR is 
##     rejected at 0.05 significance level. 
## 
## Non-Parametric Test:
## 
##     P-value for the non-parametric test of homoscedasticity:  0.01013183 
## 
##     Hypothesis of MCAR is rejected at  0.05 significance level.
##     The multivariate normality test is inconclusive.
```

We impute missing data using multiple imputation (m=50) and we aggregate the imputations by averaging.

```
# compute 50 imputations by PMM
odc.df.filter.mice<-mice(odc.df.filter, m=50, method='pmm', printFlag = F)
odc.df.multi.completed<-complete(odc.df.filter.mice, 'all', include = F)

# Aggregate the imputations by average
odc.df.multi.completed<-lapply(odc.df.multi.completed, as.matrix)
odc.df.completed<-apply(simplify2array(odc.df.multi.completed), 1:2, mean)

naniar::vis_miss(as.data.frame(odc.df.completed))
```

## PCA

Perform linear PCA with centered and unit variance scaled data

```
pca_data<-odc.df.completed
pca<-prcomp(pca_data, center = T, scale. = T)

# calculate the standardizes loadings from the PCA solution using the package
original_loadings<-syndRomics::stand_loadings(pca, pca_data)

write.csv(original_loadings,'Case1 original loadings.csv')
```

## Component selection

We perform a permutation test with P 10000 to test for significance of VAF on the first 5 PCs. As a result, we select 3 components for subsequent analysis (see paper).

```
per.VAF<-syndRomics::permut_pc_test(pca, pca_data, P=3000, ndim=5, statistic = 'VAF')

# Plot the original VAF vs. the permuted VAF
plot(per.VAF, plot_type = "VAF", ndim=1:5, plot_resample = T)
```

```
ggsave('Case1 permut VAF plot.png', width = 2.5, height = 3)

# Results of the permutation test
per.VAF$results
```

```
##       original       mean     ci_low    ci_high       pvalue  adj.p.value
## PC1 0.32913839 0.09114659 0.08421194 0.10019746 0.0003332223 0.0005553704
## PC2 0.18311886 0.08381594 0.07839324 0.08981362 0.0003332223 0.0005553704
## PC3 0.09876146 0.07829352 0.07374716 0.08341742 0.0003332223 0.0005553704
## PC4 0.07523476 0.07355731 0.06944152 0.07785066 0.2155948017 0.2694935022
## PC5 0.06253618 0.06934534 0.06558236 0.07328982 1.0000000000 1.0000000000
```

```
write.csv(per.VAF$results,'Case1 permut VAF results.csv')
```

## Component interpretation

Here we calculate the permutation test of the loadings using the permV methods (see paper for details on interpretation)

```
# Loading permutation test on 3000 resamples of the selected first 3 PCs
per.load<-syndRomics::permut_pc_test(pca, pca_data, P=3000, ndim=3, 
                                     statistic = 's.loadings', perm.method = 'permV')

# Plot the results
plot(per.load, ndim=1:3, plot_resample = T, colors=c('grey','grey','grey'), gradient_color=F, plot_cutoff=F)+
  ggtitle('s.loadings. permut. test (3000 samples)')+
  xlab(NULL)+ylab(NULL)+
  theme(plot.title.position = 'plot', plot.title = element_text(size = 10))
```

```
ggsave('Case1 permuted loadings.png', width = 6.5, height = 3.5)

# Results table
per.load$results
```

```
## # A tibble: 54 x 8
## # Groups:   component [3]
##    Variables     component original      mean ci_low ci_high  pvalue adj.p.value
##    <chr>         <chr>        <dbl>     <dbl>  <dbl>   <dbl>   <dbl>       <dbl>
##  1 BBB_FergTrans PC1        0.492    0.00455  -0.179   0.194 3.33e-4    0.000428
##  2 BBB_FergTrans PC2       -0.646   -0.00790  -0.236   0.228 3.33e-4    0.000750
##  3 BBB_FergTrans PC3        0.0347   0.00116  -0.341   0.344 8.57e-1    0.907   
##  4 ForelimbOpen~ PC1        0.513    0.00287  -0.176   0.188 3.33e-4    0.000428
##  5 ForelimbOpen~ PC2       -0.320   -0.000889 -0.221   0.225 4.00e-3    0.00654 
##  6 ForelimbOpen~ PC3        0.00435  0.00151  -0.347   0.322 9.80e-1    0.980   
##  7 Groom         PC1        0.377    0.00496  -0.178   0.190 3.33e-4    0.000428
##  8 Groom         PC2       -0.621   -0.00442  -0.228   0.221 3.33e-4    0.000750
##  9 Groom         PC3       -0.0516   0.00289  -0.342   0.340 7.89e-1    0.888   
## 10 LFPA          PC1        0.539    0.00305  -0.189   0.183 3.33e-4    0.000428
## # ... with 44 more rows
```

```
write.csv(per.load$results, "Case1 permutation load p3000.csv")
```

We do the same with the communalities

```
# Communalities permuation test with 3000 resamples
per_com<-permut_pc_test(pca, pca_data, P=3000, ndim=3, 
                                     statistic = 'commun', perm.method = 'permV')

# Plot the results
plot(per_com, plot_resample = T, ndim=1:3, plot_list_center=T, plot_legend=F, colors=c("grey","grey"), gradient_color=F)+
  ggtitle('Commun. permut. test (3000 samples) on 3 PCs')+
  xlab(NULL)+ylab(NULL)+
  theme(plot.title.position = 'plot', plot.title = element_text(size = 10))
```

```
ggsave('Case1 permuted communalities.png', width = 3.5, height = 3)

# Results table
per_com$results
```

```
## # A tibble: 18 x 7
##    Variables         original   mean  ci_low ci_high   pvalue adj.p.value
##    <chr>                <dbl>  <dbl>   <dbl>   <dbl>    <dbl>       <dbl>
##  1 BBB_FergTrans        0.660 0.0561 0.00390   0.178 0.000333    0.000333
##  2 ForelimbOpenField    0.366 0.0533 0.00370   0.167 0.000333    0.000333
##  3 Groom                0.530 0.0551 0.00370   0.170 0.000333    0.000333
##  4 LFPA                 0.613 0.0610 0.00373   0.196 0.000333    0.000333
##  5 LFSL                 0.568 0.0549 0.00345   0.175 0.000333    0.000333
##  6 LHPA                 0.785 0.0699 0.00416   0.226 0.000333    0.000333
##  7 LHSL                 0.791 0.0610 0.00407   0.199 0.000333    0.000333
##  8 PawPL                0.702 0.0566 0.00365   0.181 0.000333    0.000333
##  9 RFPA                 0.853 0.0569 0.00352   0.188 0.000333    0.000333
## 10 RFSL                 0.591 0.0554 0.00390   0.180 0.000333    0.000333
## 11 RHPA                 0.808 0.0590 0.00464   0.184 0.000333    0.000333
## 12 RHSL                 0.882 0.0581 0.00394   0.186 0.000333    0.000333
## 13 StepDistLF           0.710 0.0541 0.00400   0.173 0.000333    0.000333
## 14 StepDistLH           0.460 0.0654 0.00423   0.203 0.000333    0.000333
## 15 StepDistRF           0.536 0.0541 0.00388   0.173 0.000333    0.000333
## 16 StepDistRH           0.274 0.0558 0.00386   0.173 0.000333    0.000333
## 17 TotalSubscore        0.405 0.0549 0.00338   0.178 0.000333    0.000333
## 18 wtChng               0.464 0.0620 0.00370   0.200 0.000333    0.000333
```

```
write.csv(per_com$results, "Case1 permutation comm p3000.csv")
```

## Component stability

Bootstrap the loadings and calculate similarity metrics for the PCs using 3000 resamples with the balanced bootstrap.

```
lb<-pc_stability(pca, pca_data, B = 3000, ndim=3, s_cut_off = 0.1, 
                              sim = 'balanced', barmap_plot = T)
#table of PC similarities
lb$PC_similarity
```

```
## $similarity_mean
## # A tibble: 3 x 6
##   PC    cc_index r_correlation   rmse s_index    s_HP
##   <chr>    <dbl>         <dbl>  <dbl>   <dbl>   <dbl>
## 1 1        0.997         0.997 0.0485   0.997 0.00292
## 2 2        0.995         0.995 0.0448   0.979 0.0584 
## 3 3        0.993         0.988 0.0398   0.959 0.188  
## 
## $similarity_ci_low
## # A tibble: 3 x 6
##   PC    cc_index r_correlation   rmse s_index   s_HP
##   <chr>    <dbl>         <dbl>  <dbl>   <dbl>  <dbl>
## 1 1        0.993         0.994 0.0293   0.971 0     
## 2 2        0.990         0.990 0.0277   0.941 0.0278
## 3 3        0.984         0.974 0.0249   0.889 0.139 
## 
## $similarity_ci_high
## # A tibble: 3 x 6
##   PC    cc_index r_correlation   rmse s_index   s_HP
##   <chr>    <dbl>         <dbl>  <dbl>   <dbl>  <dbl>
## 1 1        0.999         0.999 0.0714       1 0.0278
## 2 2        0.998         0.998 0.0682       1 0.0833
## 3 3        0.997         0.996 0.0588       1 0.222
```

```
#Plot loadings
plot(lb, ndim = 1:3, plot_resample = T, gradient_color=F, colors=c("grey", "grey","grey"), plot_cutoff=F, plot_list_center=T)+
  ggtitle('Bootstrap loadings (3000 samples)')+
  xlab(NULL)+ylab(NULL)+
  theme(plot.title.position = 'plot', plot.title = element_text(size = 10))
```

```
ggsave('Case1 boot_loads.png', width = 5.2)

# Plot communalities
plot(lb, communalities = T,ndim = 1:3, plot_list_center=T,plot_resample = T, gradient_color=F, colors=c("grey", "grey","grey"))+
  ggtitle('Bootstrap communalities (3000 samples) on 3PCs')+
  xlab(NULL)+ylab(NULL)+
  theme(plot.title.position = 'plot', plot.title = element_text(size = 10))
```

```
ggsave('Case1 boot_commun.png', width = 3)

#Save similarity metrics
write.csv(lb$PC_similarity, 'Case1 boot similarity.csv')
```

### Stability of missing data

Run PCA and extract the loadings for each imputed dataset (m=50)

```
missing_loadings<-lapply(odc.df.multi.completed, function(x){
  temp_data<-x
  temp_pca<-prcomp(temp_data, scale. = T, center = T)
  
  syndRomics:::extract_loadings(temp_pca, temp_data)
})
```

We use Procrustes rotation to rotate each loading matrix obtained from each of the 50 imputed datasets towards the parent matrix to deal with indeterminacies (see paper)

```
parent<-syndRomics:::extract_loadings(pca, pca_data)
parent$Variables<-NULL

rotated_missing_loadings<-lapply(missing_loadings, function(x){
  x$Variables<-NULL
  r<-pracma::procrustes(as.matrix(parent)[,1:3],as.matrix(x)[,1:3])
  r$P
})
```

Now we extract the similarity metrics between the loadings of the missing datasets.

```
sim_missing<-syndRomics::component_similarity(rotated_missing_loadings,
                                              ndim = 3, s_cut_off = 0.2)

sim_missing$index_mean
```

```
##   PC  cc_index r_correlation       rmse   s_index       s_HP
## 1  1 0.9989426     0.9989882 0.02617984 0.9731544 0.04779812
## 2  2 0.9970842     0.9970791 0.03203249 0.9831451 0.13334000
## 3  3 0.9783278     0.9635478 0.06221812 0.9385348 0.40110988
```

```
sim_missing$index_sd
```

```
##   PC     cc_index r_correlation        rmse    s_index       s_HP
## 1  1 0.0004431907  0.0004387768 0.005266570 0.02418484 0.02805265
## 2  2 0.0016737804  0.0016799775 0.008395056 0.01720916 0.01907064
## 3  3 0.0162470837  0.0272804614 0.021768678 0.05278185 0.03833684
```

```
write.csv(sim_missing, 'Case1 boot similarity missing.csv')
```

We can also plot the average loadings and their 95% confidence intervals

```
#Build the loading dataset
load_list<-rotated_missing_loadings
load_list<-lapply(load_list, function(x){
  x[,1:3]
})

load_df<-as.data.frame(do.call(rbind, load_list))
colnames(load_df)<-paste0("PC", 1:ncol(load_df))
load_df$Variables<-rep(rownames(original_loadings),50)
load_df$id<-rep(1:50, each=nrow(original_loadings))

load_df<-load_df%>%pivot_longer(-c(id,Variables),
                              names_to = "component", values_to = "loading")

# Summarize the loadings by average and 95%CI
results<-load_df%>%
  group_by(Variables, component)%>%
  dplyr::summarise(mean=mean(loading), ci_low=quantile(loading, (1-0.95)/2, na.rm = T),
            ci_high=quantile(loading, 1-(1-0.95)/2, na.rm = T))

# Plot the loadings and the CI using the barmap_loading() function from the package
syndRomics::barmap_loading(pca, pca_data, ndim=1:3, resample_ci = results,plot_cutoff = F,
               plot_list_center = T, gradient_color=F, colors=c("grey", "grey","grey"))+
  ggtitle("Multiple imputation (m=50) loading stability")+
  xlab(NULL)+ylab(NULL)+
  theme(plot.title.position = 'plot', plot.title = element_text(size = 10))
```

```
ggsave('barmap_missing.png', height = 4.01, width = 7.5)
```

## Component visualization

Generating and saving syndRomic plots with cutoff 0.45 and heatmaps and barmap plots with cutoff 0.21, 0.25 and 0.4 for PC1, PC2 and PC3, respectively (see paper for more details)

```
# Extract syndromic plots
splot<-syndromic_plot(pca, pca_data, cutoff = 0.45)
splot$PC1
```

```
splot$PC2
```

```
splot$PC3
```

```
# Save the plots in PDF
ggsave(plot = splot$PC1,filename = 'Case1 splot PC1.pdf', width = 9, height = 9)
ggsave(plot = splot$PC2,filename = 'Case1 splot PC2.pdf', width = 9, height = 9)
ggsave(plot = splot$PC3,filename = 'Case1 splot PC3.pdf', width = 9, height = 9)
```

```
#Heatmap plot
heatmap_loading(pca, pca_data, ndim=1:3, cutoff = c(0.21,0.25,0.4), star_values = T, text_values = F)+
  ggtitle("Standardized loadings")+
  theme(plot.title.position = 'plot', plot.title = element_text(size = 10))
```

```
ggsave('Case1 heatmap.png',height = 4.01, width = 3.2)
```

# Second case study

The data is a selected subset of variables of the TRACK-TBI PILOT dataset that was used and shared by the publication:

```
Nielson JL, Cooper SR, Yue JK, Sorani MD, Inoue T, Yuh EL, Mukherjee P, Petrossian TC, Paquette J, Lum PY, Carlsson GE, Vassar MJ, Lingsma HF, Gordon WA, Valadka AB, Okonkwo DO, Manley GT, Ferguson AR; TRACK-TBI Investigators. Uncovering precision phenotype-biomarker associations in traumatic brain injury using topological data analysis. PLoS One. 2017 Mar 3;12(3):e0169490. doi: 10.1371/journal.pone.0169490. PMID: 28257413; PMCID: PMC5336356.
```

10.1371/journal.pone.0169490

For details on the analysis, please see the manuscript.

```
# Specify the path and file name of the data from 10.1371/journal.pone.0169490.s010
plos_df<-read.csv("data/journal.pone.0169490.s010.csv")
```

## Data pre-processing

We change the variable names for clarity. The data dictionary containing information for this dataset can be found at:

10.1371/journal.pone.0169490

```
#Rename the variables for easy use
plos_df<-plos_df%>%
  rename(CT_Marshall=Marshall, CT_Rotterdam=Rotterdam,
         CT_brain_pathology=CT_INTRACRANIAL_FINAL,
         CT_skull_FX=SKULLFX, CT_skullbase_FX=SKULLBASEFX,
         CT_facial_FX=FACIALFX, CT_EDH=EDH_FINAL, CT_SDH=SDH_FINAL,
         CT_SAH=SAH_FINAL, CT_contusion=CONTUSION_FINAL,
         CT_midlineshift=MIDLINESHIFT_FINAL,
         CT_cisterncomp=CISTERNCOMP_FINAL,
         PTSD_diagnosis_6mo=PTSD_6mo,
         GOSE_3mo=GOSE_OverallScore3M, GOSE_6mo=GOSE_OverallScore6M,
         WAIS_PSI_6mo=WAIS_PSI_Composite_6mo,
         CVLT_short_6mo=CVLTShortDelayCuedRecallStandardScore_6mo,
         CVLT_long_6mo=CVLTLongDelayCuedRecallStandardScore_6mo,
         SNP_COMT=rs4680, SNP_DRD2=rs6277, SNP_PARP1=rs3219119, 
         SNP_ANKK1_Gly318Arg=rs11604671, SNP_ANKK1_Gly442Arg=rs4938016,
         SNP_ANKK1_Glu713Lys=rs1800497)%>%
  relocate(PTSD_diagnosis_6mo, .before=GOSE_3mo)
```

## Missing data analysis

```
set.seed(500)

# Shadow missing plot
vis_miss(plos_df[,-1])+
  theme(plot.margin = unit(c(3,5,3,3), 'mm'),
        legend.position = 'right')
```

```
ggsave('Case2 missing shadow.png', width = 7, height = 4)

# Upset plot
png('Case2 missing upset.png', units = 'in', width = 7, height = 3.5, res = 300)
  naniar::gg_miss_upset(plos_df,nsets=20, mb.ratio=c(0.4,0.6))
dev.off()
```

```
## png 
##   2
```

The MR\_result variable has a lot of missigness, and we will drop it for this analysis. We also subtract the patient identifier.

```
plos_df<-plos_df%>%select(-PatientNum, -MR_result)
```

We transform the variables into their type for further analysis. Specifically, all CT and SNP, as well as PTSD and GOSE are categorical and we represent them as a factor in R

```
factor_vars<-colnames(plos_df)[!colnames(plos_df)%in%c("PatientNum", "CVLT_short_6mo","CVLT_long_6mo","WAIS_PSI_6mo")]

plos_df<-plos_df%>%
  mutate_at(.vars = factor_vars, .funs=factor)%>%
  mutate(WAIS_PSI_6mo=as.numeric(WAIS_PSI_6mo))
```

Now we test whether the data is missing complete at random (MCAR).

```
plos_df<-as.matrix(apply(plos_df, 2, as.numeric))
plos_df%>% 
  TestMCARNormality()
```

```
## Call:
## TestMCARNormality(data = .)
## 
## Number of Patterns:  13 
## 
## Total number of cases used in the analysis:  548 
## 
##  Pattern(s) used:
##            CT_Marshall   CT_Rotterdam   CT_brain_pathology   CT_skull_FX
## group.1              1              1                    1             1
## group.2              1              1                    1             1
## group.3              1              1                    1             1
## group.4              1              1                    1             1
## group.5              1              1                    1             1
## group.6              1              1                    1             1
## group.7              1              1                    1             1
## group.8              1              1                    1             1
## group.9              1              1                    1             1
## group.10             1              1                    1             1
## group.11             1              1                    1             1
## group.12             1              1                    1             1
## group.13             1              1                    1             1
##            CT_skullbase_FX   CT_facial_FX   CT_EDH   CT_SDH   CT_SAH
## group.1                  1              1        1        1        1
## group.2                  1              1        1        1        1
## group.3                  1              1        1        1        1
## group.4                  1              1        1        1        1
## group.5                  1              1        1        1        1
## group.6                  1              1        1        1        1
## group.7                  1              1        1        1        1
## group.8                  1              1        1        1        1
## group.9                  1              1        1        1        1
## group.10                 1              1        1        1        1
## group.11                 1              1        1        1        1
## group.12                 1              1        1        1        1
## group.13                 1              1        1        1        1
##            CT_contusion   CT_midlineshift   CT_cisterncomp   PTSD_diagnosis_6mo
## group.1               1                 1                1                   NA
## group.2               1                 1                1                   NA
## group.3               1                 1                1                    1
## group.4               1                 1                1                   NA
## group.5               1                 1                1                    1
## group.6               1                 1                1                   NA
## group.7               1                 1                1                   NA
## group.8               1                 1                1                   NA
## group.9               1                 1                1                    1
## group.10              1                 1                1                    1
## group.11              1                 1                1                    1
## group.12              1                 1                1                    1
## group.13              1                 1                1                    1
##            GOSE_3mo   GOSE_6mo   WAIS_PSI_6mo   CVLT_short_6mo   CVLT_long_6mo
## group.1          NA         NA             NA               NA              NA
## group.2          NA         NA             NA               NA              NA
## group.3           1          1              1                1               1
## group.4           1         NA             NA               NA              NA
## group.5           1          1              1                1               1
## group.6           1          1             NA               NA              NA
## group.7           1         NA             NA               NA              NA
## group.8           1          1             NA               NA              NA
## group.9           1          1              1               NA              NA
## group.10          1          1              1                1               1
## group.11          1          1             NA               NA              NA
## group.12          1          1             NA               NA              NA
## group.13         NA          1              1                1               1
##            SNP_COMT   SNP_DRD2   SNP_PARP1   SNP_ANKK1_Gly318Arg
## group.1          NA         NA          NA                    NA
## group.2           1         NA          NA                    NA
## group.3          NA         NA          NA                    NA
## group.4           1         NA          NA                    NA
## group.5           1          1           1                     1
## group.6           1          1           1                     1
## group.7          NA         NA          NA                    NA
## group.8          NA         NA          NA                    NA
## group.9           1          1           1                     1
## group.10          1          1          NA                     1
## group.11          1          1           1                     1
## group.12         NA         NA          NA                    NA
## group.13          1          1           1                     1
##            SNP_ANKK1_Gly442Arg   SNP_ANKK1_Glu713Lys   Number of cases
## group.1                     NA                    NA                42
## group.2                     NA                     1                55
## group.3                     NA                    NA                62
## group.4                     NA                     1                52
## group.5                      1                     1               194
## group.6                      1                     1                42
## group.7                     NA                    NA                19
## group.8                     NA                    NA                24
## group.9                      1                     1                11
## group.10                     1                     1                 7
## group.11                     1                     1                18
## group.12                    NA                    NA                 7
## group.13                     1                     1                15
## 
## 
##     Test of normality and Homoscedasticity:
##   -------------------------------------------
## 
## Hawkins Test:
## 
##     P-value for the Hawkins test of normality and homoscedasticity:  0 
## 
##     Either the test of multivariate normality or homoscedasticity (or both) is rejected.
##     Provided that normality can be assumed, the hypothesis of MCAR is 
##     rejected at 0.05 significance level. 
## 
## Non-Parametric Test:
## 
##     P-value for the non-parametric test of homoscedasticity:  3.004425e-08 
## 
##     Hypothesis of MCAR is rejected at  0.05 significance level.
##     The multivariate normality test is inconclusive.
```

We impute missing data using multiple imputation (m=50) and we aggregate the imputations by averaging the numeric variables or taking the mode of the categorical variables.

```
plos_df.mice<-mice(plos_df, m=50, method='pmm', printFlag = F)
plos_df.completed<-complete(plos_df.mice, 'all', include = F)

# Aggregate the dataset to the closest integer to the median
nlpca_data<-lapply(plos_df.completed, as.matrix)
nlpca_data<-apply(simplify2array(nlpca_data), 1:2, function(x){
  round(median(x))
})
```

## nonlinear PCA

Perform nonlinear PCA using *princals()*. Variables are not restricted to ordinal. A three degree polynomial is specified for the continuous transformation function for all variables.

For details on using *princals()*, we refer to:

```
De Leeuw, J., Mair, P., Groenen, P. J. F. (2017). Multivariate Analysis with Optimal Scaling.
```

and the function documentation *?princals*

```
# NLPCA with princals
nlpca<-princals(nlpca_data, ndim = ncol(nlpca_data),
                ordinal = FALSE,
                degrees = 3, knots = knotsGifi(nlpca_data, type="E"))
barmap_loading(nlpca, nlpca_data, ndim=1:5)
```

```
par(mfrow=c(1,1))
plot(nlpca, plot.type = 'screeplot')
```

```
# calculate the standardizes loadings from the PCA solution using the package
plos_df_original_loadings<-syndRomics::stand_loadings(nlpca, nlpca_data)

write.csv(plos_df_original_loadings,'Case2 original loadings.csv')
```

## Component selection

We perform a permutation test with P 500 to test for significance of VAF on the first 10 PCs. As a result, we select 3 components for subsequent analysis (see paper).

```
nlpca_per_VAF<-permut_pc_test(nlpca, nlpca_data, P=500, ndim=10, statistic = 'VAF')

# Plot the original VAF vs. the permuted VAF
plot(nlpca_per_VAF, ndim=1:8, plot_resample = T)
```

```
ggsave('Case2 permut VAF plot plos.png', width = 3, height = 4)

nlpca_per_VAF$results
```

```
##        original       mean     ci_low    ci_high      pvalue adj.p.value
## PC1  0.25799985 0.05763014 0.05523336 0.06052183 0.001996008  0.00332668
## PC2  0.10605461 0.05511054 0.05324531 0.05711313 0.001996008  0.00332668
## PC3  0.09830156 0.05322588 0.05166460 0.05484510 0.001996008  0.00332668
## PC4  0.06759141 0.05158856 0.04994000 0.05307131 0.001996008  0.00332668
## PC5  0.05860668 0.05009591 0.04873774 0.05163798 0.001996008  0.00332668
## PC6  0.05689973 0.04873401 0.04732607 0.05001104 0.001996008  0.00332668
## PC7  0.04824449 0.04743921 0.04604440 0.04882347 0.119760479  0.14970060
## PC8  0.04734903 0.04624226 0.04505540 0.04764811 0.057884232  0.08269176
## PC9  0.03949932 0.04508113 0.04398086 0.04635295 1.000000000  1.00000000
## PC10 0.03537932 0.04393092 0.04275787 0.04517811 1.000000000  1.00000000
```

```
write.csv(nlpca_per_VAF$results,'Case2 permut VAF results plos.csv')

save(nlpca_per_VAF,file="nlpca_per_VAF_plos.Rda")
```

## Component interpretation

Here we calculate the permutation test of the loadings using the permV methods (see paper for details on interpretation)

This might take several minutes to compute

```
nlpca_per_load<-syndRomics::permut_pc_test(nlpca, nlpca_data, P=500, ndim=3, 
                                     statistic = 's.loadings', perm.method = 'permV')

plot(nlpca_per_load, ndim=1:3, plot_resample = T, colors=c("grey","grey","grey"), gradient=F, cutoff=0)+
  xlab(NULL)+ylab(NULL)+
  ggtitle('Permutation loadings test (500 samples)')+
  theme(plot.title.position = 'plot', plot.title = element_text(size = 10, hjust = 0.5))
```

```
ggsave('Case2 permuted loadings, plos.png', width = 4.9)

nlpca_per_load$results
```

```
## # A tibble: 72 x 8
## # Groups:   component [3]
##    Variables      component original     mean ci_low ci_high  pvalue adj.p.value
##    <chr>          <chr>        <dbl>    <dbl>  <dbl>   <dbl>   <dbl>       <dbl>
##  1 CT_brain_path~ PC1         0.306   0.00456 -0.225   0.239 0.0140      0.0419 
##  2 CT_brain_path~ PC2         0.729   0.00434 -0.156   0.176 0.00200     0.00319
##  3 CT_brain_path~ PC3         0.129  -0.00128 -0.302   0.284 0.445       0.562  
##  4 CT_cisterncomp PC1         0.0449  0.00371 -0.246   0.262 0.721       0.721  
##  5 CT_cisterncomp PC2         0.752   0.00599 -0.162   0.182 0.00200     0.00319
##  6 CT_cisterncomp PC3        -0.231   0.00701 -0.306   0.304 0.160       0.348  
##  7 CT_contusion   PC1         0.286  -0.00563 -0.256   0.227 0.0240      0.0575 
##  8 CT_contusion   PC2         0.644   0.0101  -0.145   0.173 0.00200     0.00319
##  9 CT_contusion   PC3         0.0537 -0.00822 -0.322   0.293 0.766       0.800  
## 10 CT_EDH         PC1         0.250   0.00369 -0.251   0.243 0.0499      0.0855 
## # ... with 62 more rows
```

```
write.csv(nlpca_per_load$results, "Case2 permutation load nlpca.csv")
```

## Component stability

We bootstrap the loadings and calculate similarity metrics for the PCs using 1000 resamples with the balanced bootstrap.

```
lb_nlpca_plos<-syndRomics::pc_stability(nlpca, nlpca_data, B = 1000, 
                                        ndim=3, s_cut_off = 0.1, sim = 'balanced')
lb_nlpca_plos$PC_similarity
```

```
## $similarity_mean
## # A tibble: 3 x 6
##   PC    cc_index r_correlation   rmse s_index  s_HP
##   <chr>    <dbl>         <dbl>  <dbl>   <dbl> <dbl>
## 1 1        0.996         0.996 0.0320   0.945 0.165
## 2 2        0.997         0.997 0.0354   0.974 0.199
## 3 3        0.995         0.995 0.0297   0.974 0.201
## 
## $similarity_ci_low
## # A tibble: 3 x 6
##   PC    cc_index r_correlation   rmse s_index  s_HP
##   <chr>    <dbl>         <dbl>  <dbl>   <dbl> <dbl>
## 1 1        0.992         0.992 0.0226   0.895 0.125
## 2 2        0.995         0.994 0.0237   0.923 0.167
## 3 3        0.990         0.99  0.0204   0.919 0.167
## 
## $similarity_ci_high
## # A tibble: 3 x 6
##   PC    cc_index r_correlation   rmse s_index  s_HP
##   <chr>    <dbl>         <dbl>  <dbl>   <dbl> <dbl>
## 1 1        0.998         0.998 0.0436       1 0.208
## 2 2        0.999         0.999 0.0498       1 0.229
## 3 3        0.998         0.998 0.0407       1 0.229
```

```
#Plot the loadings
plot(lb_nlpca_plos, ndim = 1:3, plot_resample = T, gradient_color=F, colors=c("grey", "grey","grey"),plot_cutoff=F)+
  ggtitle('Bootstrap loadings (1000 samples)')+
  xlab(NULL)+ylab(NULL)+
  theme(plot.title = element_text(size = 10,hjust = 0.5), plot.title.position = "plot")
```

```
ggsave('Case2 boot_loads_plos.png', width = 4.9)

#Plot the communalities
plot(lb_nlpca_plos, communalities = T,ndim = 1:3, plot_list_center=T,plot_resample = T, gradient_color=F, colors=c("grey", "grey","grey"))+
  ggtitle('Bootstrap communalities (1000 samples) on 3 PCs')+
  theme(plot.title.position = 'plot', plot.title = element_text(size = 10))
```

```
ggsave('Case2 boot_commun_plos.png', width = 3.5)
write.csv(lb_nlpca_plos$PC_similarity, 'Case2 boot similarity_plos.csv')
```

### Stability of missing data

Helper function to run PCA and extract the loadings for each imputed dataset (m=50)

```
missing_loadings_plos<-lapply(plos_df.completed, function(x){
  temp_pca<-princals(x, ndim = ncol(x),
                ordinal = FALSE,
                degrees = 3, knots = knotsGifi(x,"E"))
  
  syndRomics:::extract_loadings(temp_pca, x)
})
```

We have to rotate each loading matrix towards the parent matrix to deal with indeterminacies (see paper)

```
parent<-syndRomics:::stand_loadings(nlpca, nlpca_data)

rotated_missing_loadings_plos<-lapply(missing_loadings_plos, function(x){
  x$Variables<-NULL
  r<-pracma::procrustes(as.matrix(parent)[,1:3],as.matrix(x)[,1:3])
  r$P
})
```

Now we extract the similarity metrics between the loadings of the missing datasets.

```
sim_missing_nlpca<-component_similarity(rotated_missing_loadings_plos,
                                        ndim = 3, s_cut_off = 0.2)

sim_missing_nlpca$index_mean
```

```
##   PC  cc_index r_correlation       rmse   s_index      s_HP
## 1  1 0.8988461     0.9038320 0.13500694 0.8530111 0.3899997
## 2  2 0.9873341     0.9850323 0.07209543 0.9248501 0.3749988
## 3  3 0.7553376     0.7577628 0.18527551 0.6533562 0.5066666
```

```
sim_missing_nlpca$index_sd
```

```
##   PC   cc_index r_correlation       rmse    s_index       s_HP
## 1  1 0.10265061    0.10085696 0.06366812 0.13010337 0.04237904
## 2  2 0.01004645    0.01238905 0.02709976 0.04678228 0.04811115
## 3  3 0.21541245    0.21231177 0.08325911 0.20452486 0.04963099
```

```
write.csv(sim_missing_nlpca, 'Case2 boot similarity missing.csv')
```

We can also plot the average loadings and their 95% confidence intervals

```
# Create the loadings data for ploting
load_list_plos<-rotated_missing_loadings_plos
load_list_plos<-lapply(load_list_plos, function(x){
  x[,1:3]
})

load_df_plos<-as.data.frame(do.call(rbind, load_list_plos))
colnames(load_df_plos)<-paste0("PC", 1:ncol(load_df_plos))
load_df_plos$Variables<-rep(colnames(nlpca_data),50)
load_df_plos$id<-rep(1:50, each=ncol(nlpca_data))

load_df_plos<-load_df_plos%>%pivot_longer(-c(id,Variables),
                              names_to = "component", values_to = "loading")

# Summarize the loadings with the average and 95%CI
results<-load_df_plos%>%
  group_by(Variables, component)%>%
  dplyr::summarise(mean=mean(loading), 
                   ci_low=quantile(loading, (1-0.95)/2, na.rm = T),
                   ci_high=quantile(loading, 1-(1-0.95)/2, na.rm = T))

# Plot the loadings and the 95%CI
barmap_loading(nlpca, nlpca_data, ndim=1:3, resample_ci = results,plot_list_center = T,colors=c("grey","grey","grey"), gradient=F, cutoff=0)+
  ggtitle("Multiple imputation (m=50) loading stability")+
  xlab(NULL)+ylab(NULL)+
  theme(plot.title.position = 'plot', plot.title = element_text(size = 10, hjust = 0.5))
```

```
ggsave('Case2 barmap_missing_plos.png', height = 4.01, width = 7.5)
```

## Component visualization

```
#Extract and save the syndromic plots
nlpca_syndromics<-syndromic_plot(nlpca, nlpca_data, cutoff = 0.4)

nlpca_syndromics$PC1
```

```
nlpca_syndromics$PC2
```

```
nlpca_syndromics$PC3
```

```
ggsave(plot = nlpca_syndromics$PC1,filename = 'Case2 splot PC1 plos.pdf', 
       width = 9, height = 9)
ggsave(plot = nlpca_syndromics$PC2,filename = 'Case2 splot PC2 plos.pdf', 
       width = 9, height = 9)
ggsave(plot = nlpca_syndromics$PC3,filename = 'Case2 splot PC3 plos.pdf', 
       width = 9, height = 9)
```

# Apendix

## Speed and precision

This script measures the computation time and the precision on the estimates of the permutation and the bootstrapping algorithms with 2 different versions of the first use case datasets. The analyzed version is considered a “small” size dataset. The “small” dataset is repeated several times to artificially increase the volume of the data and is regarded as the “large” dataset.

THIS CODE CAN TAKE A WHILE TO RUN. The script is not run at the time of rendering this file. Please make sure to set eval=TRUE in the following code chunks to run the script. Alternatively, the script can be directly evaluated in RStudio.

```
#Helper function to run the test
test_speed<-function(pca, pca_data, test, samples, k, var_id, perm="permD"){
  
  # Function taking the results of a pca and the data used on the pca to run k
  # permutation test through permut_pc_test().
  # 
  # pca: pca result from prcomp function
  # pca_data: data passed to the prcomp function to run the pca
  # samples:  numeric vector of number of permutation samples to run
  # test: Either "permutation" or "bootstrap"
  # k:  number of replicates per permutation sample
  # var_id: variable number to return the results from
  # perm: permutation method to test. Either "permD" or "permV"
  
  cat(test, "k:",k, "var_id:", var_id, "/n")
  
  #vectors to save the results on
  results_time<-vector(mode='numeric',length = k)
  results_mean<-vector(mode='numeric',length = k)
  results_m_list<-list()
  results_t_list<-list()
  pb<-progress_bar$new(total = k*10)
  
  
  #' Iterate k times for each sample to produce computation time and confident interval on the estimates
  
  for (j in 1:k){
    set.seed(j)
    for (i in 1:length(samples)){
      
      if (test=="permutation"){
        suppressMessages(s<-system.time(
        p<-permut_pc_test(pca, pca_data, ndim = 10, P=samples[i],
                          statistic="s.loading", perm.method = perm)))
      }else if (test=="bootstrap"){
        suppressMessages(s<-system.time(
        p<-pc_stability(pca, pca_data, ndim = 3, communalities = F,
                        test_similarity = F,ci_type = "perc",B = samples[i])))
      }
      
      results_time[i]<-s[3]
      results_mean[i]<-p$results$mean[var_id]
      
      pb$tick()
    }
    results_t_list[[j]]<-results_time
    results_m_list[[j]]<-results_mean
  }
  
  return(list(results_t_list, results_m_list))
}

#number of resamples to test
samples<-c(10,25,50,100,250,500,750,1000,1500, 2000)

# construct the large dataset
pca_data_large<-matrix(rep(odc.df.completed,30), ncol = 54)
colnames(pca_data_large)<-rep(colnames(odc.df.filter),3)

# Run the PCA on the large dataset
pca_large<-prcomp(pca_data_large, center = T, scale. = T)
```

### Permutation

```
# Run the different speed and precision test
small_permut_test_7<-test_speed(pca, pca_data, test="permutation", samples, 10, 7)
small_permut_test_5<-test_speed(pca, pca_data, test="permutation", samples, 10, 5)
large_permut_test_7<-test_speed(pca_large, pca_data_large, test="permutation",
                                samples, 10, 7)
large_permut_test_5<-test_speed(pca_large, pca_data_large, test="permutation",
                                samples, 10, 5)
```

**Plot speed and precision test**

```
# Create the datafrem with the results of the test for ploting
df.plot_perm<-data.frame(mean=c(unlist(small_permut_test_7[[2]]),
                                  unlist(small_permut_test_5[[2]]),
                                  unlist(large_permut_test_7[[2]]),
                                  unlist(large_permut_test_5[[2]])),
                    n_sample=rep(samples, 40),
                    time=c(unlist(small_permut_test_7[[1]]),
                                  unlist(small_permut_test_5[[1]]),
                                  unlist(large_permut_test_7[[1]]),
                                  unlist(large_permut_test_5[[1]])),
                    df_size=c(rep('159x18', 200),rep('1590x54',200)),
                    loading_size=c(rep("High loading", 100), rep("Low loading", 100),
                                   rep("High loading", 100), rep("Low loading", 100)))

df.plot_perm$df_size<-factor(df.plot_perm$df_size, levels=c("159x18", "1590x54"))

df.plot_perm<-df.plot_perm%>%
  group_by(loading_size)%>%
  mutate(center_mean=scale(mean, center = T, scale = F))

ggplot(df.plot_perm, aes(log10(n_sample),mean))+
  stat_summary(fun = 'mean', geom='line', color='red')+
  stat_summary(fun = 'mean', geom='point', color='red')+
  stat_summary(fun.data = mean_cl_normal, geom='errorbar', color='red')+
  theme_minimal(base_size = 9)+
  xlab('P size (log10)')+
  ylab('Var 1 loading in PC1')+
  geom_hline(yintercept = 0, linetype="dashed")+
  facet_grid(df_size~loading_size)

ggsave('sample size p perm.png',height = 3, width = 3)

ggplot(df.plot_perm, aes(n_sample, time, color=df_size, group=df_size))+
  stat_summary(fun= 'mean', geom='line')+
  geom_jitter(alpha=0.3)+
  scale_color_manual(values=c('steelblue', 'orange'))+
  theme_minimal(base_size = 9)+
  xlab('P size')+
  ylab('Computing time (s)')+
  theme(legend.position = c(0.3,0.85))+
  labs(color='Data size')

ggsave('sample size P time perm.png', height = 3, width = 1.7)

# Create the dataframe with the confidence interval and its width for ploting
df.plot.CI_perm<-df.plot_perm%>%
  group_by(n_sample, df_size, loading_size)%>%
  summarise(sd=sd(mean),
            mean=mean(abs(mean)),
            ci_low=mean-2.26*sd,
            ci_high=mean+2.26*sd,
            per_CIwidth=abs(ci_high-ci_low))

ggplot(df.plot.CI_perm, aes(n_sample,per_CIwidth, color=loading_size))+
  geom_line()+
  geom_point()+
  theme_minimal(base_size = 9)+
  scale_color_manual(values=c('green3', 'purple1'))+
  xlab('P size')+
  ylab('95% Confidence Interval width')+
  facet_grid(~df_size)+
  theme(legend.position = c(0.8,0.8))+
  labs(color = "Loading size")

ggsave('sample CI width perm.png',height = 3, width = 3)
```

### Bootstrapping

```
small_boot_test_7<-test_speed(pca, pca_data, test="bootstrap", samples, 10, 7)
small_boot_test_5<-test_speed(pca, pca_data, test="bootstrap", samples, 10, 5)
large_boot_test_7<-test_speed(pca_large, pca_data_large, test="bootstrap",
                              samples, 10, 7)
large_boot_test_5<-test_speed(pca_large, pca_data_large, test="bootstrap",
                              samples, 10, 5)
```

**Plot speed and precision test**

```
# Create the datafrem with the results of the test for ploting
df.plot<-data.frame(mean=c(unlist(small_boot_test_7[[2]]),
                                  unlist(small_boot_test_5[[2]]),
                                  unlist(large_boot_test_7[[2]]),
                                  unlist(large_boot_test_5[[2]])),
                    n_sample=rep(samples, 40),
                    time=c(unlist(small_boot_test_7[[1]]),
                                  unlist(small_boot_test_5[[1]]),
                                  unlist(large_boot_test_7[[1]]),
                                  unlist(large_boot_test_5[[1]])),
                    df_size=c(rep('159x18', 200),rep('1590x54',200)),
                    loading_size=c(rep("High loading", 100), rep("Low loading", 100),
                                   rep("High loading", 100), rep("Low loading", 100)))

df.plot$df_size<-factor(df.plot$df_size, levels=c("159x18", "1590x54"))

df.plot<-df.plot%>%
  group_by(loading_size)%>%
  mutate(center_mean=scale(mean, center = T, scale = F))

ggplot(df.plot, aes(log10(n_sample),center_mean))+
  stat_summary(fun = 'mean', geom='line', color='red')+
  stat_summary(fun = 'mean', geom='point', color='red')+
  stat_summary(fun.data = mean_cl_normal, geom='errorbar', color='red')+
  theme_minimal(base_size = 9)+
  xlab('B size (log10)')+
  ylab('Var 1 loading in PC1 (centered to mean)')+
  facet_grid(df_size~loading_size)+
  geom_hline(yintercept = 0, linetype="dashed")

ggsave('sample size B.png',height = 3, width = 3)

ggplot(df.plot, aes(n_sample, time, color=df_size, group=df_size))+
  stat_summary(fun= 'mean', geom='line')+
  geom_jitter(alpha=0.3)+
  scale_color_manual(values=c('steelblue', 'orange'))+
  theme_minimal(base_size = 9)+
  xlab('B size')+
  ylab('Computing time (s)')+
  theme(legend.position = c(0.32,0.85))+
  labs(color='Data size')

ggsave('sample size B time.png', height = 3, width = 1.7)

# Create the dataframe with the confidence interval and its width for ploting
df.plot.CI<-df.plot%>%
  group_by(n_sample, df_size, loading_size)%>%
  summarise(sd=sd(mean),
            mean=mean(abs(mean)),
            ci_low=mean-2.26*sd,
            ci_high=mean+2.26*sd,
            per_CIwidth=abs(ci_high-ci_low))

ggplot(df.plot.CI, aes(n_sample,per_CIwidth, color=loading_size))+
  geom_line()+
  geom_point()+
  theme_minimal(base_size = 9)+
  scale_color_manual(values=c('green3', 'purple1'))+
  xlab('B size')+
  ylab('95% Confidence Interval width')+
  facet_grid(~df_size)+
  theme(legend.position = c(0.8,0.8))+
  labs(color = "Loading size")

ggsave('sample CI width B.png',height = 3, width = 3)
```

## Session information

```
sessionInfo()
```

```
## R version 4.0.2 (2020-06-22)
## Platform: x86_64-w64-mingw32/x64 (64-bit)
## Running under: Windows 10 x64 (build 19041)
## 
## Matrix products: default
## 
## locale:
## [1] LC_COLLATE=English_United States.1252 
## [2] LC_CTYPE=English_United States.1252   
## [3] LC_MONETARY=English_United States.1252
## [4] LC_NUMERIC=C                          
## [5] LC_TIME=English_United States.1252    
## 
## attached base packages:
## [1] stats     graphics  grDevices utils     datasets  methods   base     
## 
## other attached packages:
##  [1] Gifi_0.3-9            naniar_0.6.0          MissMech_1.0.2       
##  [4] mice_3.11.0           forcats_0.5.0         purrr_0.3.4          
##  [7] readr_1.3.1           tibble_3.0.3          tidyverse_1.3.0      
## [10] syndRomics_0.0.2.9000 progress_1.2.2        tidyr_1.1.2          
## [13] stringr_1.4.0         ggplot2_3.3.2         dplyr_1.0.2          
## 
## loaded via a namespace (and not attached):
##  [1] ggrepel_0.8.2     Rcpp_1.0.5        lubridate_1.7.9.2 ggnewscale_0.4.3 
##  [5] lattice_0.20-41   prettyunits_1.1.1 assertthat_0.2.1  digest_0.6.25    
##  [9] R6_2.4.1          cellranger_1.1.0  backports_1.1.9   reprex_0.3.0     
## [13] visdat_0.5.3      evaluate_0.14     pracma_2.2.9      httr_1.4.2       
## [17] pillar_1.4.6      rlang_0.4.7       readxl_1.3.1      rstudioapi_0.11  
## [21] blob_1.2.1        rmarkdown_2.3     labeling_0.3      munsell_0.5.0    
## [25] broom_0.7.0       compiler_4.0.2    modelr_0.1.8      xfun_0.17        
## [29] pkgconfig_2.0.3   htmltools_0.5.0   tidyselect_1.1.0  codetools_0.2-16 
## [33] fansi_0.4.1       crayon_1.3.4      dbplyr_1.4.4      withr_2.2.0      
## [37] grid_4.0.2        jsonlite_1.7.1    gtable_0.3.0      lifecycle_0.2.0  
## [41] DBI_1.1.0         magrittr_1.5      scales_1.1.1      cli_2.0.2        
## [45] stringi_1.5.3     farver_2.0.3      fs_1.5.0          xml2_1.3.2       
## [49] ellipsis_0.3.1    generics_0.1.0    vctrs_0.3.4       boot_1.3-25      
## [53] tools_4.0.2       glue_1.4.2        hms_0.5.3         yaml_2.2.1       
## [57] colorspace_1.4-1  rvest_0.3.6       knitr_1.29        haven_2.3.1
```
